# Using UAV-Based Temporal Spectral Indices to Dissect Changes in the Stay-Green Trait in Wheat

**DOI:** 10.34133/plantphenomics.0171

**Published:** 2024-04-30

**Authors:** Rui Yu, Xiaofeng Cao, Jia Liu, Ruiqi Nie, Chuanliang Zhang, Meng Yuan, Yanchuan Huang, Xinzhe Liu, Weijun Zheng, Changfa Wang, Tingting Wu, Baofeng Su, Zhensheng Kang, Qingdong Zeng, Dejun Han, Jianhui Wu

**Affiliations:** ^1^ College of Agronomy, Northwest A&F University, Yangling, Shaanxi 712100, China.; ^2^ State Key Laboratory of Crop Stress Resistance and High-Efficiency Production, Northwest A&F University, Yangling, Shaanxi 712100, China.; ^3^ College of Mechanical and Electronic Engineering, Northwest A&F University, Yangling, Shaanxi 712100, China.; ^4^ College of Plant Protection, Northwest A&F University, Yangling, Shaanxi 712100, China.

## Abstract

Stay-green (SG) in wheat is a beneficial trait that increases yield and stress tolerance. However, conventional phenotyping techniques limited the understanding of its genetic basis. Spectral indices (SIs) as non-destructive tools to evaluate crop temporal senescence provide an alternative strategy. Here, we applied SIs to monitor the senescence dynamics of 565 diverse wheat accessions from anthesis to maturation stages over 2 field seasons. Four SIs (normalized difference vegetation index, green normalized difference vegetation index, normalized difference red edge index, and optimized soil-adjusted vegetation index) were normalized to develop relative stay-green scores (RSGS) as the SG indicators. An RSGS-based genome-wide association study identified 47 high-confidence quantitative trait loci (QTL) harboring 3,079 single-nucleotide polymorphisms associated with SG and 1,085 corresponding candidate genes. Among them, 15 QTL overlapped or were adjacent to known SG-related QTL/genes, while the remaining QTL were novel. Notably, a set of favorable haplotypes of SG-related candidate genes such as *TraesCS2A03G1081100*, *TracesCS6B03G0356400*, and *TracesCS2B03G1299500* are increasing following the Green Revolution, further validating the feasibility of the pipeline. This study provided a valuable reference for further quantitative SG and genetic research in diverse wheat panels.

## Introduction

Wheat varieties with high and stable yields are required to meet the demands of a growing global population and a changing climate [1]. Senescence in wheat is a highly complex developmental process that progresses from flowering until harvest ripeness and significantly contributes to the final yield [2,3]. To select cultivars with optimal senescence process, stay-green (SG) has been defined by breeders to characterize senescent dynamics of different cultivars. High SG refers to a strong capacity to maintain canopy vigor after anthesis, which delays senescence and prolongs photosynthetic activity during grain filling [4,5] and are preferable selected in breeding to achieve higher yield and stronger stress tolerance [5–7].

Hence, considerable efforts have been made in the past few years to understand the genetic basis. Some SG-related genes/quantitative trait loci (QTL) have been identified through linkage mapping in populations of recombinant inbred lines (RILs), doubled haploid (DH), and multi-reference nested association mapping (MR-NAM) in wheat [8–10]. For example, the senescence-promoting factor *NAM-B1* was explored by QTL mapping, where a reduction in RNA levels of the multiple *NAM* homologs delayed senescence [11]. *TaARF15-A1* was identified as a negative regulator of senescence in wheat; it suppressed the expression of *TaNAM-1* via protein–protein interaction and competition with *TaMYC2* for binding to its promoter to regulate senescence [12]. Successful genetic research on complex traits such as SG requires high-quality genomic and phenotypic data. Recent advancements in genome sequencing have greatly facilitated the access to high-quality genomics data, highlighting that the exploration of SG-related genetic basis is mainly hindered by the lack of proper quantification of SG [13].

Breeders and geneticists are familiar with the term “grew yellow” to qualitatively describe the extent of SG in wheat. They often use the traditional method of visual scoring to evaluate SG or senescence, which is a low-efficiency and labor-intensive process [14]. In recent years, the development and application of high-throughput plant phenotype technology have benefited various plants, including remote sensing detection by unmanned aerial vehicles (UAVs) and satellites for field phenotype detection [15]. However, UAV-based images have a higher resolution and a lower cost and are not affected by high-altitude clouds, making them more effective for accurate detection of phenotypes [16,17]. To accurately quantify the complex comprehensive trait of SG, it is essential to measure the changes in green leaf area and chlorophyll content of the entire plant. However, this is not feasible at the population level. Instead, UAV-based temporal spectral indices (SIs) can reflect the changes in greenness of plant populations and have been widely used as an alternative to traditional methods for acquiring phenotypes to evaluate SG traits [18,19]. Several recent research projects have developed phenotypic pipelines to quantify SG phenotype and simultaneously identified novel genetic loci related SG by QTL mapping [10,18,19]. For example, some researchers have used logistic functions using temporal normalized difference vegetation index (NDVI) to calculate SG parameters. DH and MR-NAM populations in wheat identified 43 and 65 SG-related QTL, respectively [10,20]. However, most of these pipelines are only suitable for small genotype panels with relatively consistent phenology such as heading or anthesis date. The difference in phenology is usually significant among individuals in diverse wheat panels, especially when comparisons are based on a single stage of development or absolute senescence responses of individual accessions. Therefore, an effective pipeline encompassing multi-stage SG trait quantification needs to be explored to address these issues.

The green normalized difference vegetation index (GNDVI), NDVI, normalized difference red edge index (NDRE), and optimized soil-adjusted vegetation index (OSAVI) have been proven to effectively reflect the chlorophyll content and green biomass of plant populations and have successfully been applied in the evaluation of plant greenness [21–25]. Additionally, the effectiveness of these 4 SIs in evaluating wheat SG has also been confirmed in our previous study [26]. This study selected these 4 SIs to build a relative stay-green scores (RSGS) pipeline that used the thermal time after anthesis to calibrate the incomparability of phenotypes caused by inconsistent flowering time. It quantified wheat SG in 4 critical stages (Stage 1 [S1]: the milk-ripe stage, S2: the transitional stage from milk-ripe to mealy ripe stage, S3: the middle mealy ripe stage, and S4: the late mealy ripe stage) after anthesis related to yield formation. The temporal SG traits were used to dynamically track the senescence of a diverse wheat panel, solving the limitation of single-stage development state or absolute senescence reaction in quantitative SG. In addition, by combining genome-wide association studies (GWAS), we identified new SG-related genes that were selected during domestication and in modern breeding. The favorable haplotypes that were more intensively selected in different wheat regions further validated the reliability of this phenotyping pipeline.

## Materials and Methods

### Plant materials

A panel of 565 diverse wheat accessions, including Chinese landraces (CL), introduced modern cultivars (IMC), modern Chinese cultivars (MCC), and breeding lines from our previously collected approximately 5,000 accessions, was used for phenotyping [27]. Detailed descriptions of the accessions are provided in Table S1. This panel underwent genotyping with the 660 K single-nucleotide polymorphism (SNP) array (Beijing CapitalBio Technology Company, http://www.capitalbiotech.com) and was used for genome-wide association mapping. Further information regarding the quality control of genotype data in this panel was reported in Wu et al. [27]. A second panel consisting of 584 available genome re-sequences, based on published data from the website http://wheatomics.sdau.edu.cn/, was primarily used for candidate gene-based association analysis [28]. A pair of wheat cultivars, Shaannong 235 and Shannong 15 with the contrary phenotype (SG/non-SG), were used for RNA-seq analysis.

### Field experimentation

**Fig. 1.**
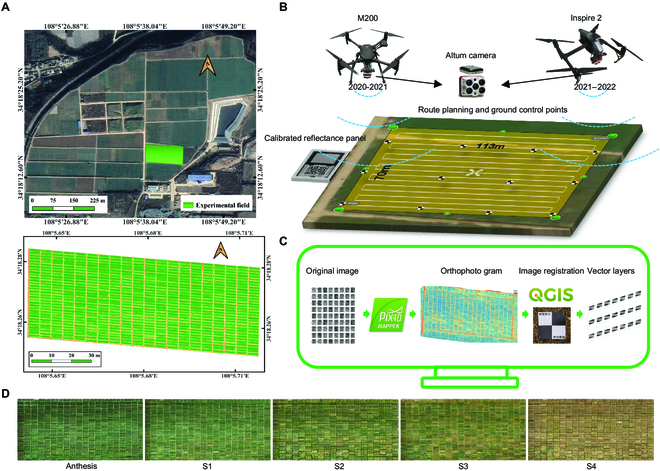
Experimental design. (A) Location of Cao Xinzhuang Experimental Farm (above) and the experimental field (below). (B) M200 and Inspire 2 UAVs, GPS and DLS sensors, Micasense Altum camera, calibrated reflectance panel, and UAV route planning. (C) Image mosaic based on Pix4D mapper and schematic diagram of wheat plot image clipping based on QGIS. (D) The schematic diagram of the 5 stages of wheat senescence process (Anthesis, S1: the milk-ripe stage, S2: the transitional stage from milk-ripe to mealy ripe stage, S3: the middle mealy ripe stage, and S4: the late mealy ripe stage).

The panel of 565 diverse wheat accessions was planted at Cao Xinzhuang Experimental Farm in Yangling, Shaanxi province, China (34.31°N, 108.10°E) during the 2 cropping seasons (2020 to 2022) (Fig. 1A). The experiments were arranged in an augmented design to correct the final yield, with 16 blocks and 5 control plots repeated in each block by ACBD-R [29]. Each plot measured 6 m^2^ (6 × 5 m rows with 20 cm spacing) and had a plant density of 2.7 million per hectare. Reflectance measurements were taken from 50% anthesis until harvest ripeness. Meteorological data obtained from the National General Weather Station in Yangling were used to calculate the thermal time of the samples during each test period (Fig. S1). The chlorophyll content of 248 flag leaf samples was measured using the SPAD-502 Plus device (Konica Minolta Holdings, Inc.) during the 4 key stages (S1 to S4) of wheat grain formation in 2021 to 2022, and an average value was computed from 5 measurements. The yield was determined at physiological maturity of the plots.

### Acquisition of temporal multispectral images

During the 2020 to 2021 and 2021 to 2022 seasons, the MicaSense Altum camera (MicaSense, USA) was mounted on a DJI Matrice 200 UAV and a DJI Inspire 2 UAV (DJ-Innovations Technology, China) (Fig. 1B) to collect data, respectively. The camera encompassed 5 narrow spectral bands, and the specific information for each band is shown in Table 1. The spectral bands had a spatial resolution of 2,064 × 1,544 (pixel) and a ground sample distance (GSD) of 5.28 cm per pixel at a height of 120 m. A Downwelling Light Sensor 2 was used to correct for changes in ambient light changes during flight. Twelve ground control points were uniformly fixed in the field prior to the first flight (Fig. 1B). A Global Positioning System (GPS) sensor recorded the geographic location information of the images. A calibrated reflectance panel (CRP) was photographed before each flight to calibrate the reflectance of the bands (Fig. 1B). The Pix4D capturer software (https://www.pix4d.com/product/pix4dcapturer) was used to plan data collection. The flight path followed the direction of the wheat rows (east-to-west) for multiple passes to capture the imagery (Fig. 1B). The flight altitude above the wheat canopy was 26 to 27 m, and the flight speed remained at 1.5 to 1.7 m/s. The camera captured images at a nadir angle, and continuously took images at 1-s intervals during the flight, with an overlap of 85% to 90% in both the forward and side directions. Temporal images were acquired at intervals of 2 to 4 days under full sunshine between 12:30 and 13:00. UAV spectral data were obtained from anthesis to harvest ripeness in both the 2020 to 2021 and 2021 to 2022 seasons, respectively.

**Table 1.** Spectral specifications of the MicaSense Altum camera

Spectral band	Band abbreviation	Central wavelength	Band width	Reflectance calibration coefficients
Blue	B	475 nm	32 nm	0.542
Green	G	560 nm	27 nm	0.542
Red	R	668 nm	14 nm	0.539
Red edge	RE	717 nm	12 nm	0.533
Near infrared	NIR	842 nm	57 nm	0.537

### Image processing and SI extraction

The captured images from each flight were used in the structure-from-motion method to generate reflectivity orthophoto maps for each band using Pix4D Mapper 4.5.6 software (Fig. 1C). Irradiance compensation and radiometric calibration were applied using the irradiance data recorded by the downward light sensor (DLS) and the reflectivity calibration coefficients from the CRP during the image mosaic pre-processing. The geographical deviations of the orthophoto maps for different flight dates were calibrated based on ground control points using Quantum GIS (QGIS) 3.10.10 software (https://www.qgis.org/en/site/) (Fig. 1C), and the orthophoto obtained from the first flight was used as a reference for geographic calibration. Shapefiles were created for each plot using QGIS 3.10.10 software, and the plot numbers were stored in the shapefiles (Fig. 1C). To eliminate the influence of background noise on the data, an image threshold segmentation method was used based on the distribution of the NDVI obtained during the first flight, selecting 0.6 as the threshold for image segmentation and generating a mask layer. The reflectance images of various spectral bands were batch-cropped using Python, resulting in images of different types of plots (Fig. 1C). Four SIs (NDRE, NDVI, GNDVI, and OSAVI; the definitions are described in Table S2), which were relatively sensitive to changes in canopy chlorophyll concentration, green biomass, and green leaf area [24,30–32], were extracted using MATLAB2020 (https://ww2.mathworks.cn/).

### Quantification of SG traits

The RSGS of individual genotypes was calculated to quantify the multistage SG trait at 4 senescence stages using temporal SIs. The milk-ripe stage (S1) (FS 11.1), transitional stage from milk-ripe to mealy ripe stage (S2) (FS 11.1 to 11.2), middle mealy ripe stage (S3) (FS 11.2), and late mealy ripe stage (S4) (FS 11.3) were critical physiological periods for grain formation during wheat grain filling, especially during S1, S3, and S4 (Fig. 1D). At the arrival of the next stage, the rapid movement of nutrients in the grain formation led to a noticeable change in the overall canopy greenness of wheat, serving as a reference stage for RSGS (Fig. 1D). The “SG” status at anthesis, when vegetative growth was completely stopped and reproductive growth was fully underway, was used as the maximum SG reference (scored as 100). The relative senescence score (RSS) during each stage (S*i*, where *i* = 1, 2, 3, 4), denoted as RSS_S*i*_, was calculated using Eq. 1. The RSGS at stage S*i* (denoted as RSGS_S*i*_), was calculated using Eq. 2 after the flowering of all samples.

$${\mathrm{RSS}}_{Si}=\frac{\left({\text{Index}}_{\text{anthesis}}-{\text{Index}}_{Si}\right)}{{\text{Index}}_{\text{anthesis}}}\times 100$$ (1)

$${\text{RSGS}}_{Si}=100-{\mathrm{RSS}}_{Si}$$ (2)

where Index_anthesis_ and Index_S*i*_ represent the index values of samples at anthesis and a specific S*i* stage, respectively.

The study included samples with different flowering periods, categorized as early flowering (EF), middle flowering (MF), and late flowering (LF). To ensure comparability of the phenotypes among EF, LF, and MF samples, the post-anthesis accumulated temperatures (AT) were considered. The AT was calculated as the sum of daily mean temperatures based on the fact that post-anthesis senescence in wheat is primarily influenced by temperature [33]. Equation 3 was used to enhance the comparability of phenotypes, and the multistage AT values of EF, LF, and MF samples during the 2 growing seasons are provided in Table S3.

$${\text{RSGS}}_{Si\_\text{crorrection}}=100-k_{i}\times {\mathrm{RSS}}_{Si}$$ (3)

where *k_i_* represents the correction factor for inconsistent flowering periods of samples at stage S*i*; *k_i_* is the ratio of AT of MF samples at stage S*i* to the AT of EF or LF samples at stage S*i*. Furthermore, the RSGS of MF samples and the corrected RSGS of EF and LF samples, calculated by each index at S*i* stage, were labeled as SG_indexS*i*_ for ease of subsequent descriptions.

### Phenotypic analysis of SG traits

Firstly, the broad-sense heritability (*H*^2^) was calculated as Eq. 4.

$$H^{2}=\frac{\delta_{G}^{2}}{\left(\delta_{G}^{2}+\delta_{e}^{2}/n\right)}$$ (4)

where $\delta_{G}^{2}\;$ represents the genetic variance, $\delta_{e}^{2}$ represents the residual error variance, and *n* represents the number of environments. The values of $\delta_{G}^{2}$ and $\delta_{e}^{2}\;$ were estimated through analysis of variance (ANOVA) using the lmer function in the lme4 package within the R environment (http://www.R-project.org/). Subsequently, Pearson correlation coefficients (PCCs) and linear regression analysis were performed using the R program.

### Population structure, principal component analysis, and linkage disequilibrium analysis

Population structure was assessed using unlinked markers (*r*^2^ = 0) in STURCTURE 2.3.4 [34]. The model was applied without using prior population information, and the most likely number of subpopulations was determined using a previously described method [34]. Principal component analysis (PCA) was conducted with the GCTA software used for assessing population structure [35]. Linkage disequilibrium (LD) analysis for the whole genome and A, B, and D genomes was performed using PLINK software [27]. LD decay distance was obtained by constructing *r*^2^ values against SNP loci and fitting these data to a locally weighted polynomial regression curve using the R program (http://www.R-project.org/).

### GWAS, fixation index, and cross-population composite likelihood ratio analysis

GWAS was performed using the GEMMA software with a univariate mixed linear model (MLM) [36]. The suggested threshold for *P* values ranged from 1.38 × 10^−4^ to 6.02 × 10^−4^ for each chromosome, and a uniform value of *P* = 2.60 × 10^−4^ was adopted as the threshold criterion for genome-wide significance. QTL were defined as continuously significant markers within a distance of 5 Mb. The identified GWAS loci were compared with previously identified QTL based on their physical locations in the Chinese Spring reference genome v2.1. For previously reported SG genes/QTL, confidence intervals were generated using the closest flanking markers. Whether the loci identified in the GWAS were novel depended on the haplotype block interval.

The population differentiation fixation index (*F*st) and cross-population composite likelihood ratio (XP-CLR) score between wheat genotypes released pre- and post-1970 were calculated for each 100-kb window across the entire wheat genome using the VCFtools software (v.4.0). The parameters in the program were set as follows: “--fst -window-size 1,000,000 --fst-window-step 100,000 --weir-fst-pop” and “--size 1,000 --step 2,000 --ld 0.95”. To detect genes from the selection, the ranking of selection sweeps was based on decreasing *F*st and XP-CLR scores, and the top 5% of regions were chosen as selective sweeps.

### RNA-seq

The RNA-seq experiment included mock and heat-stressed flag leaves of Shaanong 253 and Shanong 15. When the wheat plants reached 10 days after anthesis, the mock group was treated with normal growth conditions in the greenhouse: 22°C for 16 h and 18°C for 8 h. Meanwhile, the test group was subjected to heat stress conditions for 3 consecutive days: 38°C for 8 h and 24°C for 16 h. Subsequently, flag leaf tissues were collected from Shaanong 253 and Shanong 15 at 0, 24, 48, and 72 h, using 3 independent biological replicates. The leaf tissues from the 3 biological replicates were combined in equal amounts and sent to BGI Genomics, BGI-SHENZHEN (https://www.genomics.cn/), for RNA isolation, RNA library construction, and RNA-seq. Quality control, alignment, fragment counting, and TPM (transcripts per million kb calculation) analysis were performed on the RNA-seq data, as referenced in Yi et al. [37].

### Identification of candidate genes

Based on IWGSC RefSeq v2.1 gene annotations (http://wheatomics.sdau.edu.cn/), high confidence (HC) genes located within QTL were used for candidate gene analysis. Candidate genes or homologous amino acid sequences for SG in other plant species were downloaded from https://bigd.big.ac.cn/lsd/ to compare with potential wheat genes (*E* value <10^−5^). Gene Ontology (GO) functional enrichment was performed using http://wheat.cau.edu.cn/TGT/ [38] and visualization was carried out using clusterProfiler (v3.18.1) in the R package. Genes involved in the senescence pathway were selected as potential candidate genes. Simultaneously, the expression levels of the candidate genes were further confirmed. Finally, candidate gene-based association analysis was conducted using the MLM method in GAPIT 3.0, with a threshold for significance set at *P* = 2.60 × 10^−4^. We followed 3 principles for candidate gene analysis within the confidence interval: (a) If the QTL region contained known genes controlling SG, those genes were considered high-priority candidate genes; (b) if the QTL regions contained wheat homologs associated with genes regulating SG or senescence in other species, those genes were considered priority candidate genes; (c) if the QTL regions did not contain either of these types, the QTL were considered to be new loci.

## Results

### Genetic diversity of wheat panel

**Fig. 2.**
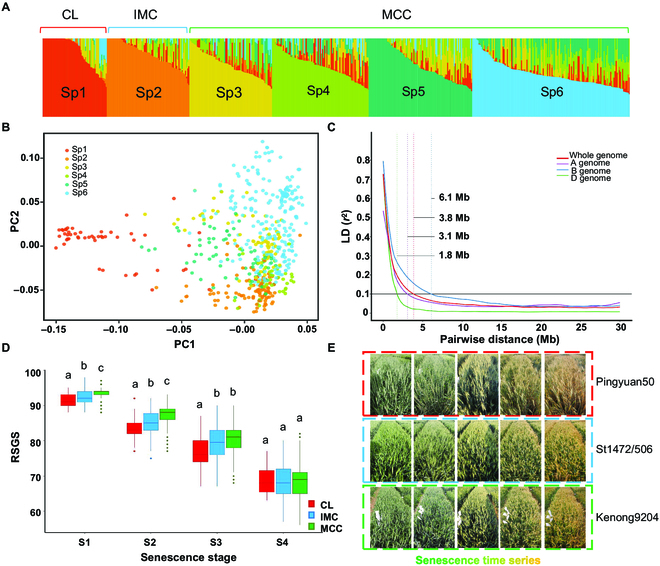
Genetic structure of the wheat panels. (A) Population structure of the panels. Red, orange, yellow, light green, dark green, and blue, respectively, represent sub-populations (Sp)1 to 6, where Sp1 denotes the Chinese landraces (CL) group (highlighted in red), Sp2 represents the introduced modern cultivars (IMC) group (highlighted in blue), and Sp3 to Sp6 correspond to the modern Chinese cultivars (MCC) group (highlighted in green). (B) Principal component analysis (PCA) of wheat panels. (C) Linkage disequilibrium (LD) decay over physical distances. It portrays the pairwise single-nucleotide polymorphism LD (*r*^2^) values as a function of inter-marker map distance (in megabase) within the 3 subgenomes. (D) The SG scores of CL, IMC, and MCC based on SG_GNDVI_ in 2022 indicate the significant differences (*P* < 0.05, LSD test). (E) The SG phenotype of Pingyuan 50 (CL), St1472/506 (IMC), and Kenong 9204 (MCC) during the senescence time series.

Population structure and PCA based on Bayesian clustering identified 6 sub-populations (Sp) (Fig. 2A and B). The LD (*r*^2^), analyzed through pairwise comparisons of 361,293 SNPs, decayed to the critical *r*^2^ value (0.1) and was estimated at approximately 3.8 Mb for the whole genome (Fig. 2C). The LD decay was faster in the D genome, with a decay of 1.8 Mb, followed by the A and B genomes at 3.1 Mb and 6.1 Mb, respectively (Fig. 2C). The faster LD decay in the D genome was related to relatively less artificial selection after its incorporation into common wheat. Geographical origin, historical timing, and temperature/light characteristics were major factors determining the diversity groups in this panel. In terms of population structure, Sp1 (Fig. 2A) mainly consisted of CL; Sp2 mainly contained IMC; Sp3 mainly included Chinese spring cultivars (CSC); Sp4 predominantly included mixed winter cultivars (MWC); Sp5 comprised Chinese winter cultivars, mostly pre-2000 (CHWC); and Sp6 mainly included CHWC grown after 2000 (post-2000). Sp3 to Sp6 were combined as MCC (Fig. 2A). During the process of senescence, CL, IMC, and MCC showed significant differences in SG phenotype in S1, S2, and S3 (Fig. 2D and 2E). Moreover, the RSGS in varieties released post-1970 were higher than those released in pre-1970 (Fig. S2).

### Evaluation of phenotypic performance

**Fig. 3.**
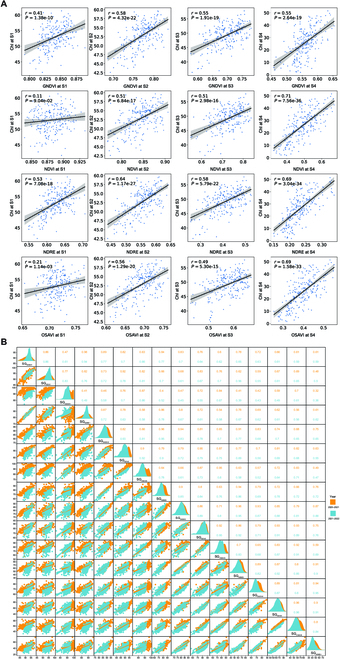
(A) Pearson correlation coefficients (PCCs) of GNDVI, NDVI, NDRE, and OSAVI with SPAD-measured flag leaf chlorophyll content (ChI) at 4 stages (S1 to S4) during the 2021 to 2022 period. The solid lines represent the fitted relationships obtained from regression models, and the shaded region indicates the 95% confidence interval. (B) PCCs and phenotypic distribution results of SG traits for both the 2020 to 2021 and 2021 to 2022 periods.

The average dynamics of the SIs of all genotypes during the 2020 to 2021 and 2021 to 2022 seasons are shown in Fig. S3. All SI values showed a consistent decline over the post-anthesis thermal time. The decline was initially slow but increased as physiological maturity approached. The decline rate was relatively slow during the watery ripe and milky ripe stages (post-anthesis AT <400 °C per day) due to less competition for assimilates from source to sink. However, it accelerated from the mealy ripe stage onward (post-anthesis AT >400 °C per day) as competition for assimilates increased. Additionally, research has revealed PCCs between temporal SIs and the relative reference index of chlorophyll content in the 2021 to 2022 season. PCCs between SIs and flag leaf chlorophyll content were particularly high during the late senescence period. The highest PCC (*r*) between SIs and flag leaf chlorophyll content was 0.71 (Fig. 3A), and the highest regression result *R*^2^ was 0.5 (Fig. S4), indicating that SIs could effectively detect different SG stages of wheat populations to a certain extent and robustly track the senescence process in the wheat canopy under field conditions.

**Fig. 4.**
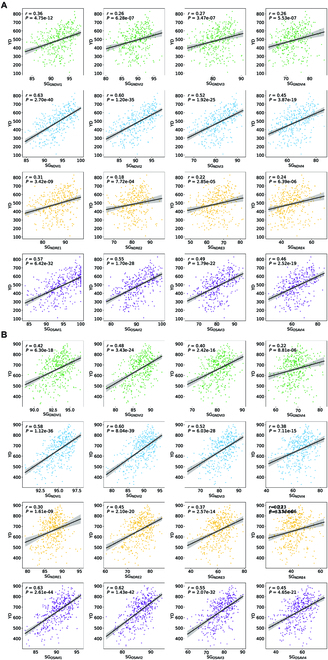
Pearson correlation coefficients (PCCs) of SG traits with yield (YD [kg/acre]) at 4 stages (S1 to S4) during the 2020 to 2021 (A) and 2021 to 2022 (B) period. Green, blue, yellow, and purple represent SG_GNDVI_, SG_NDVI_, SG_NDRE_, and SG_OSAVI_, respectively. The solid lines depict the fitted relationships derived from regression models, while the shaded region indicates the 95% confidence interval.

The quantitative results of SG traits for the wheat genotypes during both seasons are shown in Table 2, and the distribution of phenotypes for common accessions is shown in Fig. 3B and Fig. S5. The mean values of the SG traits decreased over the post-anthesis thermal time, while the standard deviations increased. The numerical range of each index varied from stage S1 to S4. For S1 and S2, the values of SG_NDRE_ were lower than those of SG_NDVI_ and SG_OSAVI_ in both years, and temporal NDRE revealed greater RSSs (>12 at S1 and >18 at S2) for the canopy during the early senescence phase in both years. The PCCs between different RSGS traits during both seasons are shown in Fig. 3B. For RSGS calculated using each index, there was generally a strong linear correlation (*r* > 0.82) between SG_index1_ and SG_index2_. Additionally, the linear relationships of SG_index3_ with SG_index2_ and SG_index4_ were also strong (*r* > 0.83) in both years. The PCCs of RSGS ranged from 0.17 to 0.73 between the 2 cropping seasons, with a mean of 0.43, indicating a relatively strong correlation. Furthermore, the PCCs between temporal SG traits and yield were established in both seasons. The results demonstrated that the temporal traits SG_NDVI_ and SG_OSAVI_ had better PCCs with yield (*r* ranged from 0.38 to 0.63) (Fig. 4A and B), and the regression analysis showed an *R*^2^ ranging from 0.15 to 0.40 (Fig. S6). The heritability (*H*^2^) of all SG traits among the 4 stages ranged from 0.31 to 0.84, with an average of 0.65 (Table S4). Among the 4 SG traits, heritability increased with the advancement of the senescence stage, while SG_OSAVI_ showed higher heritability than other SG traits across all stages. Analysis of the aforementioned results revealed a minor difference in the distribution of SG traits between both seasons, along with a strong correlation and high heritability, making them suitable for subsequent GWAS.

**Table 2.** Means and standard deviations of SG traits in different seasons

Season	SG trait	Mean ± SD			
		S1 stage	S2 stage	S3 stage	S4 stage
2020–2021	SG_NDRE_	86.76 ± 5.41	81.13 ± 5.83	65.59 ± 6.91	46.36 ± 7.50
	SG_NDVI_	94.02 ± 3.36	91.26 ± 4.02	82.21 ± 5.64	66.53 ± 8.30
	SG_GNDVI_	92.30 ± 3.20	89.49 ± 3.35	81.57 ± 4.18	73.13 ± 4.66
	SG_OSAVI_	95.12 ± 4.13	91.37 ± 5.08	81.67 ± 6.65	65.03 ± 8.76
2021–2022	SG_NDRE_	87.20 ± 2.67	74.50 ± 5.38	59.81 ± 8.02	41.60 ± 7.03
	SG_NDVI_	95.03 ± 1.55	89.03 ± 3.65	80.81 ± 6.63	63.54 ± 8.86
	SG_GNDVI_	93.18 ± 1.55	86.71 ± 3.09	79.92 ± 4.14	68.32 ± 4.59
	SG_OSAVI_	90.47 ± 2.88	86.00 ± 4.02	77.53 ± 6.73	57.04 ± 8.90

### GWAS of SG traits

**Fig. 5.**
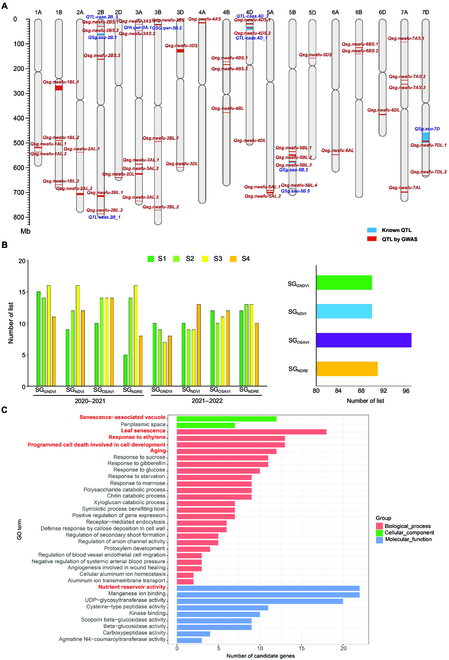
(A) Distribution of the SG QTL discovered in this study and known SG QTL on wheat chromosomes. Blue bands represent known SG QTL; red bands represent SG QTL detected by GWAS in the present study. The width of each band indicates the respective confidence interval. (B) Frequency of QTL localization for multiple SG traits across various senescence stages. (C) GO enrichment analysis of candidate genes within the SG QTL region.

GWAS was performed to explore the genetic basis of SG-related QTL in a diverse panel. The study identified 47 high-confidence QTL that contained 3,079 significant SNPs associated with the SG traits over the course of 2 years (Table S5). The *R*^2^ values for each QTL ranged from 0.1% to 26%, with a mean of 3.4% (Table S5). The distribution of QTL on chromosomes is shown in Fig. 5A, and Manhattan plots of the SG traits are listed in Figs. S7 to S10. Thirty and 32 QTL were detected in 2020 to 2021 and 2021 to 2022, respectively (Table S5), with 15 being co-located in both seasons (Table S5). Fifteen QTL either overlapped or were adjacent to previously reported QTL or genes (Table 3; Fig. 5A). For example, *Qsg.nwafu-5BL.3* was identified on Chr 5B using 4 SG traits in 2020 to 2021 and 2021 to 2022, and it overlapped the reported SG-related QTL *QSg.sau-5B.3* [8]. The SG-related gene *TaARF15* within *Qsg.nwafu-6BL.2* was detected using SG_OSAVI_ in 2020 to 2021 and SG_NDVI_, SG_OSAVI_, and SG_NDRE_ in 2021 to 2022 [12]. The number of QTL detected for each SG trait varied between years. In 2020 to 2021, SG_GNDVI_ showed the highest number of QTL, followed by SG_NDVI_, SG_OSAVI_, and SG_NDRE_ (Fig. 5B). In 2021 to 2022, SG_NDRE_ displayed the highest number of QTL, followed by SG_OSAVI_, SG_NDVI_, and SG_GNDVI_ (Fig. 5B)_._ Among the 4 SG traits, SG_OSAVI_ appeared the most frequently in the identified QTL during the 4 stages of senescence, accounting for 97 occurrences (Fig. 5B). Additionally, the number of QTL detected by the SG traits varied across multiple stages. During the 2 seasons, 7 QTL were exclusively found at stages S1 and S2, while 4 QTL were exclusively found at S3 and S4. Thirty-six QTL were found in at least 3 out of the 4 stages (Table S5).

**Table 3.** QTL overlapping or adjacent to reported SG-related QTL or genes

QTL	Chr	Physical location (Mb)	SG traits		−Log(*P*)	Known SG-related QTL or gene	Reference
			2020–2021	2021–2022			
*Qsg.nwafu-1BL.1*	1B	275.81–293.48		SG_GNDVI1_, SG_NDRE1_	4.73	*WCBP1*	[54]
*Qsg.nwafu-2BS.1*	2B	32.93–32.13	SG_GNDVI4_, SG_NDRE3_, SG_NDRE4_, SG_NDVI4_, SG_OSAVI4_		4.62	*QTL-caas.2B_5*	[18]
*Qsg.nwafu-2BS.2*	2B	71.40–71.41		SG_GNDVI1_, SG_NDVI1_, SG_NDVI2_, SG_NDRE1_	4.82	*QSg.sau-2B.1*	[8]
*Qsg.nwafu-2BL.2*	2B	795.57–795.90	SG_GNDVI2_, SG_GNDVI3_, SG_GNDVI4_, SG_NDRE2_, SG_NDRE3_, SG_NDRE4_	SG_NDRE1_	4.13	*QTL-caas.2B_1*	[18]
*Qsg.nwafu-2DL*	2D	650.27–651.57	SG_NDRE2_, SG_NDRE3_, SG_NDVI1_, SG_NDVI2_, SG_OSAVI1_, SG_OSAVI2_, SG_OSAVI3_		5.72	*SGR*	[53,55]
*Qsg.nwafu-3AS.1*	3A	18.44–19.71	SG_NDRE3_, SG_NDRE4_, SG_NDVI3_, SG_NDVI4_, SG_OSAVI4_		4.31	*QHt.qwr-3A.1*	[10,20]
*Qsg.nwafu-3BS*	3B	26.74–30.13	SG_GNDVI1_, SG_GNDVI3_, SG_GNDVI4_, SG_NDRE1_, SG_NDRE2_, SG_NDRE3_	SG_GNDVI1_, SG_GNDVI2_, SG_GNDVI3_, SG_GNDVI4_, SG_NDVI3_, SG_NDVI4_, SG_OSAVI2_, SG_OSAVI3_, SG_OSAVI4_, SG_NDRE1_, SG_NDRE2_, SG_NDRE3_, SG_NDRE4_	4.80	*QSG.qwr-3B.2*	[10,20]
*Qsg.nwafu-3DS*	3D	122.51–134.95	SG_OSAVI1_, SG_OSAVI2_, SG_OSAVI3_		4.45	*NYC1*	[53,56]
*Qsg.nwafu-4DS.1*	4D	16.57–19.87	SG_GNDVI3_, SG_GNDVI4_, SG_NDRE4_, SG_NDVI4_, SG_OSAVI1_	SG_OSAVI1_, SG_NDRE3_, SG_NDRE4_	4.43	*QTL-caas.4D_3*, *QTL.06*; *SAG39*	[18,45,57]
*Qsg.nwafu-4DS.2*	4D	38.94–39.23	SG_OSAVI3_	SG_NDRE1_, SG_NDRE3_	4.04	*QTL-caas.4D_1*	[18]
*Qsg.nwafu-5BL.2*	5B	549.72–554.78	SG_GNDVI1_, SG_GNDVI2_, SG_NDRE1_, SG_NDRE2_	SG_GNDVI2_, SG_GNDVI3_, SG_NDVI4_, SG_OSAVI4_, SG_NDRE2_, SG_NDRE3_, SG_NDRE4_	5.50	*SAG12*	[53,58]
*Qsg.nwafu-5BL.3*	5B	574.76-580.69	SG_GNDVI1_, SG_GNDVI2_, SG_GNDVI3_, SG_NDRE2_, SG_NDVI2_, SG_NDVI3_, SG_OSAVI2_, SG_OSAVI3_	SG_GNDVI1_, SG_GNDVI4_, SG_NDVI4_, SG_OSAVI3_, SG_OSAVI4_, SG_NDRE1_, SG_NDRE3_, SG_NDRE4_	4.94	*QSg.sau-5B.3*	[8]
*Qsg.nwafu-5BL.4*	5B	686.51-686.70	SG_GNDVI2_, SG_NDRE2_, SG_NDRE3_		4.23	*QSG.qwr-5B.5*	[10,20]
*Qsg.nwafu-6BS.2*	6B	146.22-146.61	SG_OSAVI1_	SG_NDVI3_, SG_NDVI4_, SG_OSAVI2_, SG_OSAVI3_, SG_OSAVI4_, SG_NDRE2_, SG_NDRE3_, SG_NDRE4_	4.92	*TaARF15*	[12]
*Qsg.nwafu-7DL.1*	7D	493.84-497.25	SG_GNDVI1_, SG_NDVI1_, SG_NDVI2_, SG_NDVI3_, SG_OSAVI2_		4.73	*QSg.sau-7D*	[8]

### Candidate genes for SG-related traits

Firstly, GO enrichment analysis was conducted on 1,085 high-confidence candidate genes located within the QTL, revealing that some of these genes were directly or indirectly involved in plant senescence. A total of 37 significantly enriched GO terms (*P* < 0.05) were identified, with the top 35 terms across the 3 GO enrichment types presented in Fig. 5C and Table S6. This analysis strongly suggested the significant association of these genes with cellular components such as senescence-associated vacuoles and biological processes including leaf senescence, ethylene response, and programmed cell death. The functions of these genes primarily relate to nutrient reservoir activity. Next, based on the 3 principles for candidate gene analysis, a superior candidate gene, *TraesCS2A03G1081100* (*D2HGDH*), was identified within *Qsg.nwafu-2AL.2*. This gene was detected by SG_GNDVI_, SG_NDRE_, SG_NDVI_, and SG_OSAVI_ in the 2020 to 2021 season and showed 72.43% homology with Arabidopsis *AT4G36400* (*D2HGDH*) at the protein level. *AT4G36400* belongs to the same network as several genes involved in β-oxidation and degradation of branched-chain amino acids in chlorophyll. This network plays a role in delaying senescence to some extent [39]. Another known gene, *TaARF15* (*TraesCS6B03G0356400*), was identified within *Qsg.nwafu-6BS.2* and has recently been associated with the JA-mediated wheat senescence pathway [12]. In terms of biological processes, *TraesCS2B03G1299500* (*WRKY70*) within *Qsg.nwafu-2BL.1* belongs to GO:0010150 and participates in leaf senescence downstream of the developmental process pathways. Based on its role as a negative regulator of leaf senescence in Arabidopsis [40], *WRKY70* was considered a key candidate gene for SG. Moreover, all 3 candidate genes showed increased expression levels in Shaannong 253 (SG) compared to Shannong 15 (non-SG) at different time points after heat stress (Fig. S11).

### Selection of favorable SG haplotypes in breeding

**Fig. 6.**
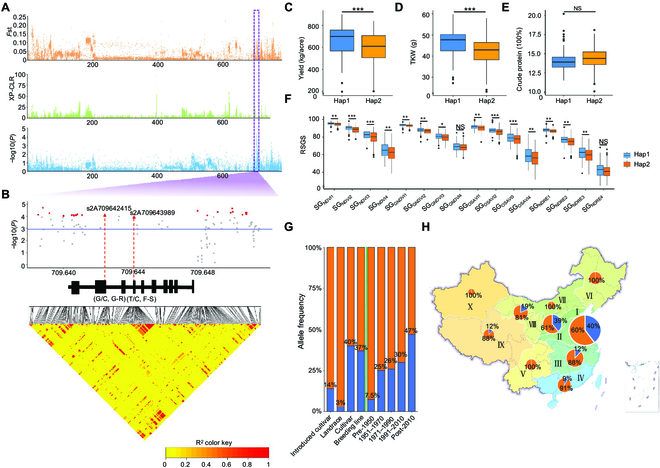
Variation in *TraesCS2A03G1081100*. (A) The *F*st, XP-CLR, and their associated significant signals on chromosome 2A. The purple rectangle is the associated signal position in chromosome 2A. (B) Local Manhattan plot (top) and LD heat map (bottom) surrounding *TraesCS2A03G1081100*. The purple line shows the significance threshold (−log10[*P* value] =3.0). Red triangles represent 2 variations in *TraesCS6B03G0356400*. Red color highlights strong LD with significant variation. (C to F) Yield, thousand kernel weight, crude protein, and RSGS for the 2 haplotypes during the 2021 to 2022 period. The *P* values were calculated using 2-tailed *t* tests (*P < 0.05; **P < 0.01; ***P < 0.001; NS, not significant). (G) The percentages of the 2 haplotypes among different wheat groups (left of the green line) and breeding periods (right of the green line). Hap1 is represented in blue, and Hap2 is represented in orange. (H) Percentages of the 2 haplotypes in different wheat zones. The size of each circle corresponds to the number of accessions; Hap1 is shown in blue, and Hap2 is represented in orange.

The priority gene *TraesCS2A03G1081100* (*D2HGDH*), identified in this study, was selected for validation in breeding. To detect indirect selection of SG-related loci or genes during modern breeding, we divided the panels into pre- and post-1970 groups (Fig. S2). *F*st and XP-CLR were then used to identify putatively selected regions during modern breeding. *Qsg.nwafu-2AL.2*, where the priority gene *TracesCS2A03G1081100* is located, showed strong selection signals (Fig. 6A). Two notable SNPs, *s2A709642415* and *s2A709643989*, were identified in *TraesCS2A03G1081100* (*D2HGDH*), leading to missense variations at positions 135 bp (G/C, Gly/Arg) and 252 bp (T/C, Phe/Ser) in the coding sequence, respectively (Fig. 6B). Significant differences were observed in dynamic SG, thousand-kernel weight (TKW), and yield between the Hap1 (C-C) group (86 accessions) and Hap2 (G-T) group (158 accessions) (Fig. 6C, D, and F), while no significant difference was found in crude protein content (Fig. 6E). Hap1 was identified as the favorable haplotype. An analysis of the 2 major haplotypes, Hap1 (195 accessions) and Hap2 (603 accessions), among the 798 worldwide wheat accessions released before 2020 revealed that the frequency of Hap1 increased from 7.5% before 1950 to 47% after 2010, indicating strong selection (Table S7; Fig. 6G). The frequencies of Hap1 in wheat regions I, II, and III were 39%, 40%, and 12%, respectively (Table S7; Fig. 6H). Furthermore, the frequencies of favorable SG haplotypes were higher in MCCs and breeding lines compared to the CL group (Fig. 6G).

Analysis of the reported gene *TaARF15-B1* (*TracesCS6B03G0356400*) (Table S8 and Fig. S12) and the priority gene *TracesCS2B03G1299500* (*WRKY70*) (Table S9 and Fig. S13) in breeding revealed a pattern similar to that of *TracesCS2A03G1081100*, with significant selection signals observed in the genome. As the years of breeding increased, the frequency of SG haplotypes in varieties (lines) and the IMC group was higher than that in the CL group. Additionally, the frequency of SG haplotypes was higher in the main wheat-producing regions (I and II) of China.

## Discussion

### High-throughput phenotyping of SG traits in the wheat diversity panel

Systematically evaluating the SG attributes at the canopy level of the diverse panel of wheat and investigating the genetic basis of these traits would facilitate the improvement of wheat breeding. This study developed a pipeline for assessing SG traits in the wheat diversity panel using UAV-based temporal SIs such as NDVI, GNDVI, NDRE, and OSAVI. These 4 SIs have demonstrated potential in detecting crop senescence at the canopy level and ascertaining wheat senescence patterns [10,41–43]. Multiple SIs sensitive to population greenness were used to obtain RSGS at several key senescence stages of wheat. Additionally, the post-anthesis AT of samples was incorporated to enhance the comparability of phenotypic data, addressing inconsistencies in flowering. This pipeline overcame limitations of traditional methods [6,8,9] and effectively captured the SG phenotype by expanding the evaluation beyond populations with consistent flowering periods, such as RILs [18,20]. The stratification of the population genetic structure first highlighted the diversity within our test population, as evident from the distinct RSGS observed in CL, IMC, and MCC. Notably, these groups had similar segmentation patterns in terms of RSGS and key agronomic traits, such as TKW; MCC demonstrated superiority over IMC and CL [44]. Secondly, the relatively strong correlation observed between these SIs, SG traits, and chlorophyll content of flag leaves, as well as their association with yield, indicated the reliability of our method. Among the SIs, NDRE displayed a higher correlation with flag leaf chlorophyll compared to others due to the high sensitivity of red edges to chlorophyll [23]. While a few low correlations were observed, it was likely that they were not indicative of simple linear relationships, especially concerning changes in greenness at the leaf and canopy levels. Nevertheless, our pipeline provided valuable insights into the SG attributes at the canopy level, which could be used for other crops with similar senescence patterns. However, the presence of a notable discrepancy in the florescence within a panel poses challenges for our pipeline. Incorporating a denser temporal sampling approach and considering additional environmental factors would help construct a model capable of assessing panels with a wide range of flowering periods.

### SG QTL detection in the wheat diversity panel

GWAS was performed using the phenotype data collected through the improved pipeline. Out of the 47 QTL identified, 30% coincided with or were tightly close to known SG-related QTL/genes, such as *TaARF15-B1* [12] and *SAG12* [45], suggesting that the pipeline was effective in phenotyping for SG. Additionally, GO enrichment analysis of the candidate genes within the QTL further supported the validity of high-throughput phenotyping. Notably, the frequency of the QTL implied that SG_OSAVI_ had a greater potential for evaluating SG since it could effectively reduce soil reflectivity interference caused by plant decay during late wheat senescence [24]. However, evaluating SG and conducting genetic studies that integrate multiple SG traits have been the most comprehensive approaches. This is because accurately capturing the characteristics of wheat senescence that occur in a temporally coordinated manner, as well as locating SG QTL across multiple stages or different environments, the combination of multiple SG traits can compensate for the lack of information obtained from a single SG trait [46]. Besides, this study used the wheat diverse panel to identify a larger number of SG QTL compared to using parental populations [8,18]. This highlighted the capacity of the wheat diverse panel to investigate the genetic basis of SG. Therefore, this pipeline could be applied in the future to screen SG germplasm from large-scale germplasm resources. SG germplasm is crucial for overcoming the current breeding bottleneck and conducting a more extensive range of GWAS studies to deeply understand the genetic basis of SG [47,48].

### Utilization of SG haplotypes in modern breeding

Previous studies on the genetics of SG traits in wheat have primarily focused on identifying QTL [8,10,49]. In this study, by combining genetic analysis, we observed that the SG candidate genes identified here had signs of selection in past wheat breeding. We found that the wheat germplasm displayed variability in SG traits prior to and after 1970, particularly during the S4 phase (Fig. S2). In fact, selection signals indicated a gradual increase in the frequency of some key SG-related genes or QTL after 1970 (Fig. S14). For instance, the haplotype *TracesCS2A03G1081100* Hap1, associated with higher yield and SG, experienced a clear increase during 1951 to 1970, known as the Green Revolution when new varieties were developed from introduced germplasm or crosses of local and foreign genotypes (Fig. 6G and Figs. S12G and S13G) [5,50]. This cross-effect became more pronounced after 1970 due to the breeding cycle (Fig. 6G and Figs. S12G and S13G). SG-related favorable haplotypes also showed distinct patterns in given regions. The frequency of favorable SG-related haplotypes was higher in humid and semi-humid areas compared to arid and semi-arid regions, especially in wheat regions I and II [12]. In our work, we observed a similar pattern for *TracesCS2A03G1081100*, where the frequency of the favorable SG-related haplotype Hap1 was higher in wheat regions I and II compared to other regions (Fig. 6H). It could be attributed to the fact that breeders in these regions have preferentially selected haplotypes with beneficial SG traits, as they generally lead to higher production levels. Furthermore, since this haplotype is relatively rare in modern germplasm, it could be a target for future selection.

### An SG haplotype for yield–quality trade-off

Studies have suggested that SG negatively affects protein content and nitrogen conversion, especially in leguminous crops [2,51]. Senescence is a dynamic process involving well-coordinated degradation and remobilization that influences both crop productivity and quality. The degradation of chloroplasts and chlorophyll in green tissues serves as the primary nitrogen source for grains [52,53]. In wheat, more than 50% of nitrogen accumulation in grains can be attributed to chlorophyll degradation in flag leaves [2]. Therefore, physiologists and breeders are interested in understanding how the onset and pace of senescence are regulated to synchronize the relationship between the sink and the source. This synchronization is crucial to ensure that nitrogen accumulation remains unaffected while simultaneously enhancing yield. Interestingly, in this study, Hap1 of *TraesCS2A03G1081100* showed high TKW and yield, without affecting crude protein contents (Fig. 6C to F). Based on the above, it could be inferred that the negative relationship between SG and protein content can potentially be overcome by incorporating suitable genes through breeding.

## Data Availability

The genotype and phenotype data presented in this study are available at the website https://github.com/zengqd/PopulationGenetics/tree/main/Wheat/StayGreen.
