# Supplementary material for: Using UAV-Based Temporal Spectral Indices to Dissect Changes in the Stay-Green Trait in Wheat
Source: Plant Phenomics. 2024 Apr 30;6:0171. doi: 10.34133/plantphenomics.0171 (PMC11062509; doi:10.34133/plantphenomics.0171)
Supplement: Supplementary 1 — Figs. S1 to S14 Tables S1 to S9 [file plantphenomics.0171.f1.zip › Supplementary Figs.docx]

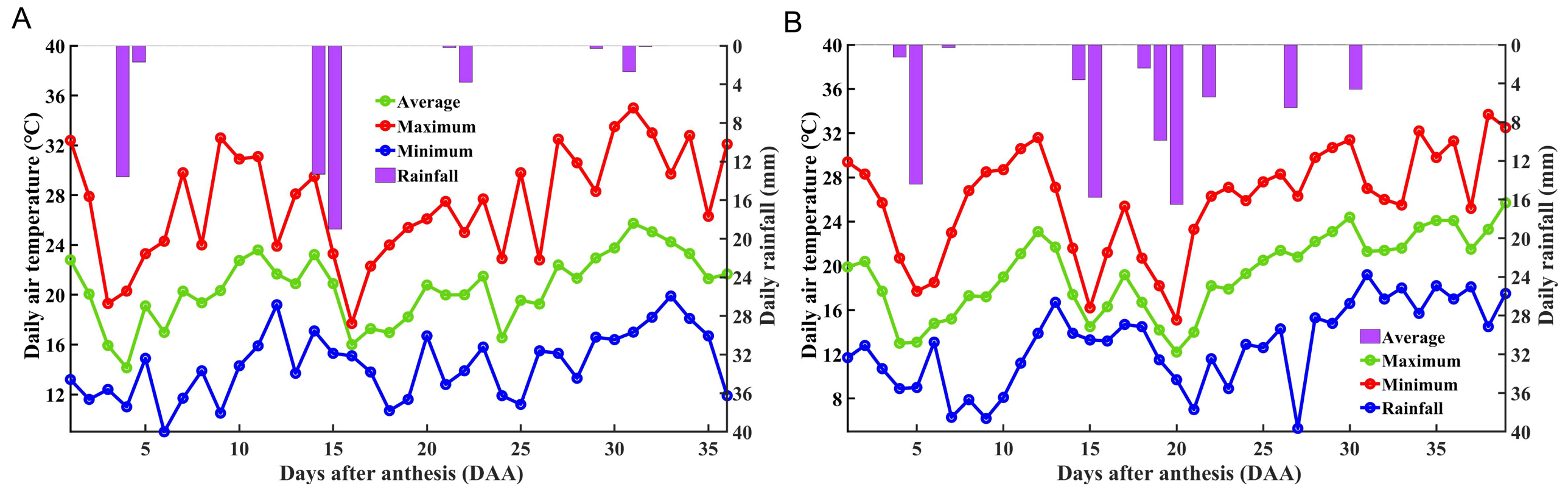


**Fig. S1** Daily air temperature and rainfall post-anthesis during the 2020-2021 (A) and 2021-2022 (B) growing seasons.


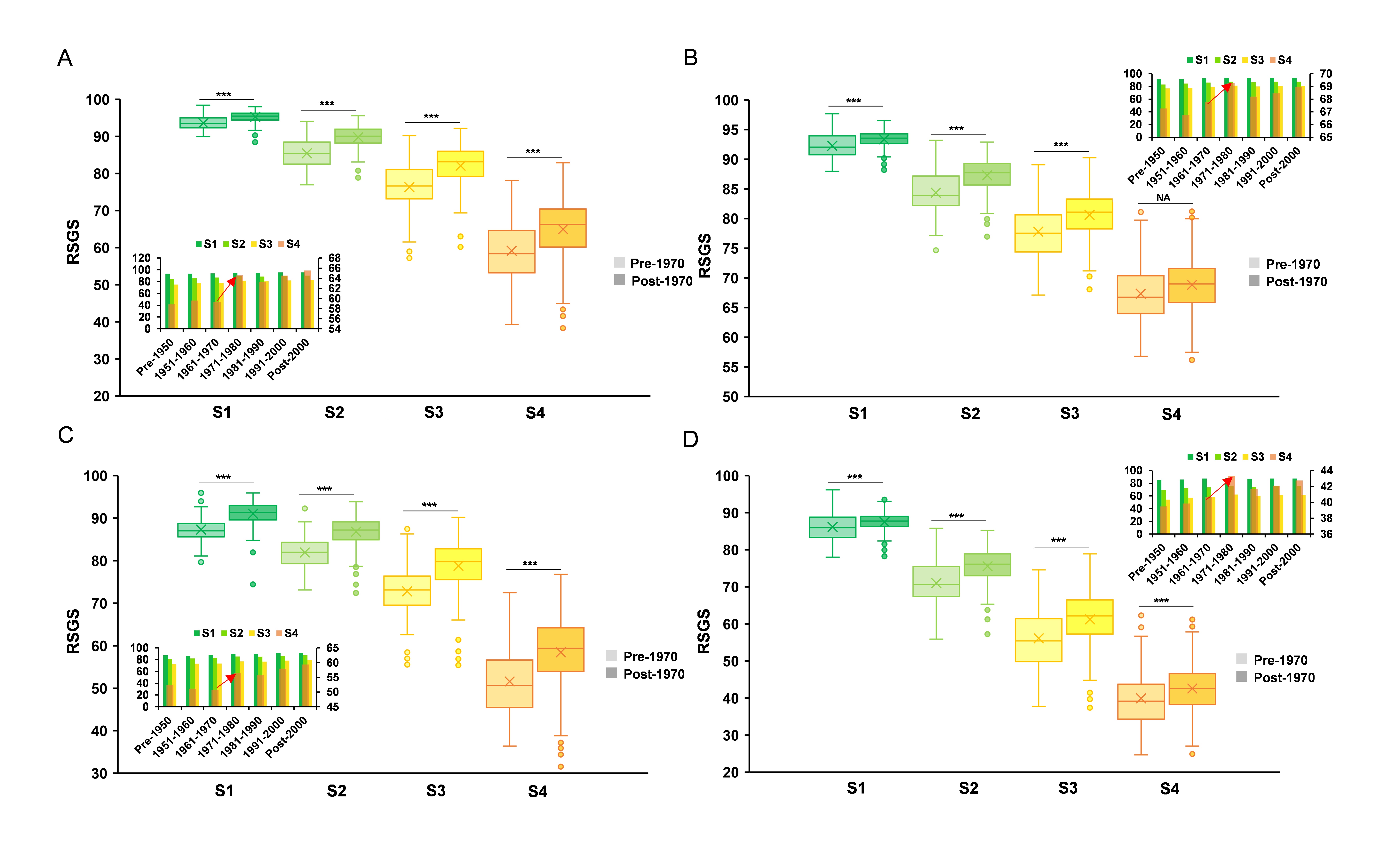


**Fig. S****2** Relative stay green scores (RSGS) in 2021-2022 panel lines released pre- and post-1970. (A) RSGS based on SG_NDVI_ and average RSGS in the panel at different release times are shown in the lower left corner. (B) RSGS based on SG_GNDVI_ and average RSGS in the wheat panel at different release times are in the upper right corner. (C) RSGS based on SG_OSAVI_ and average RSGS in the wheat panel at different release times are shown in the lower left corner. (D) RSGS based on SG_NDRE_ and average RSGS in the wheat panel at different release times are shown in the upper right corner.





**Fig. S3** Dynamics of fitted temporal SIs in 2020-2021 (A) and 2021-2022 (B). Lines represent the mean values of the indices, and shaded areas represent standard deviations (SD).

.


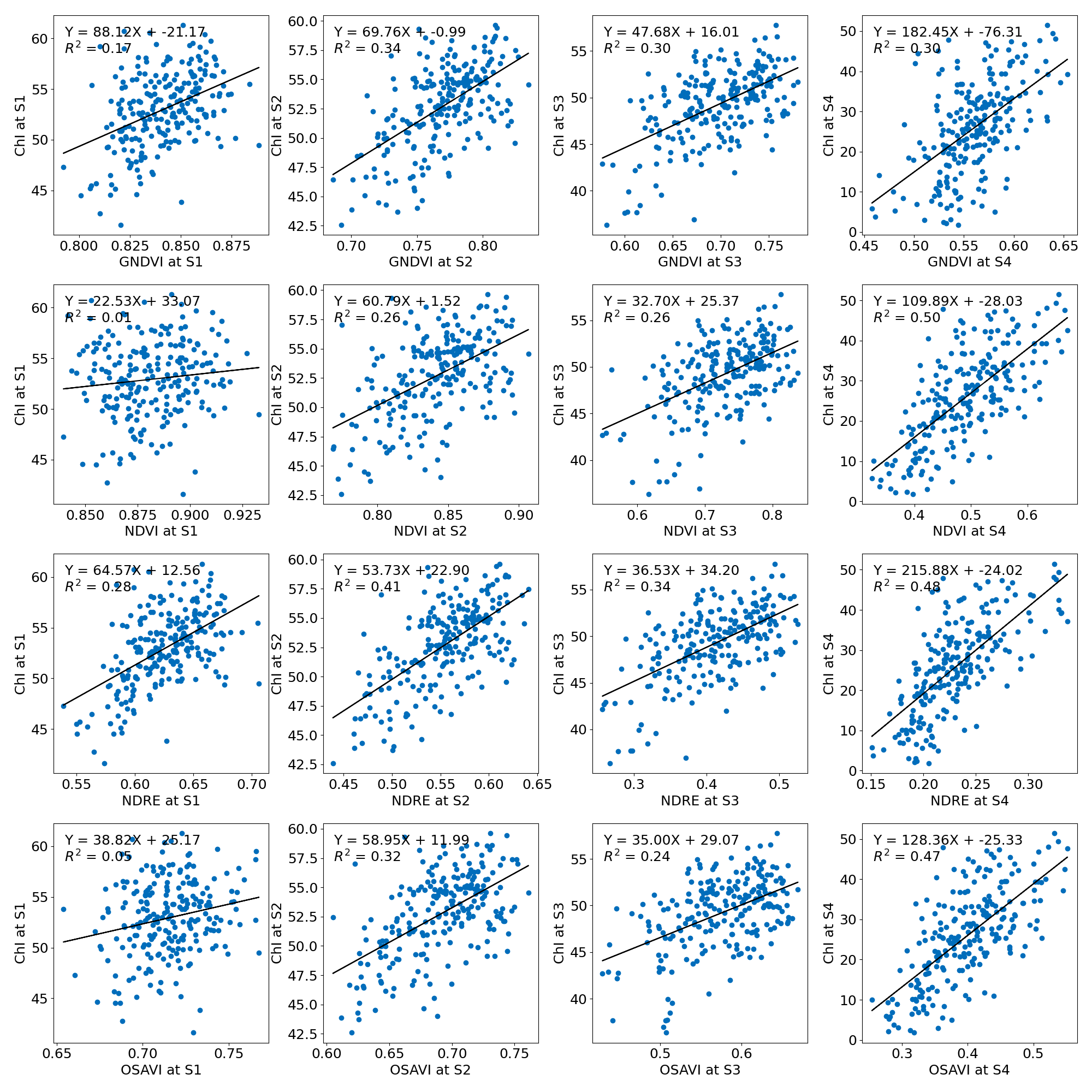


**Fig. S4** Regression results of GNDVI, NDVI, NDRE, and OSAVI with SPAD-measured flag leaf chlorophyll content at four stages (S1 to S4) in the 2021-2022.


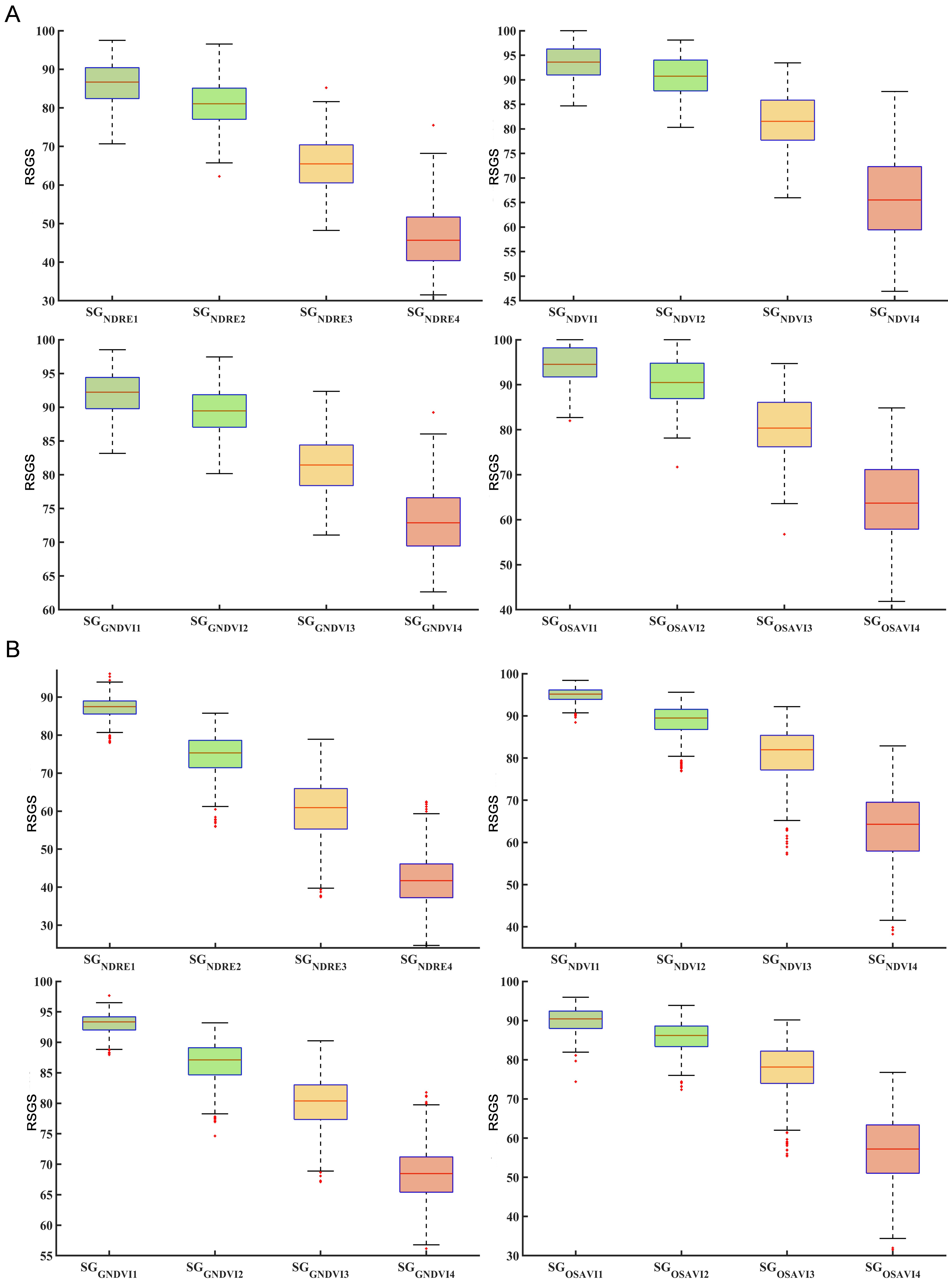


**Fig. S5** SG_NDRE_, SG_NDVI_, SG_GNDVI_, and SG_OSAVI_ at different stages in 2021-2022 (A) and 2021-2022 (B).


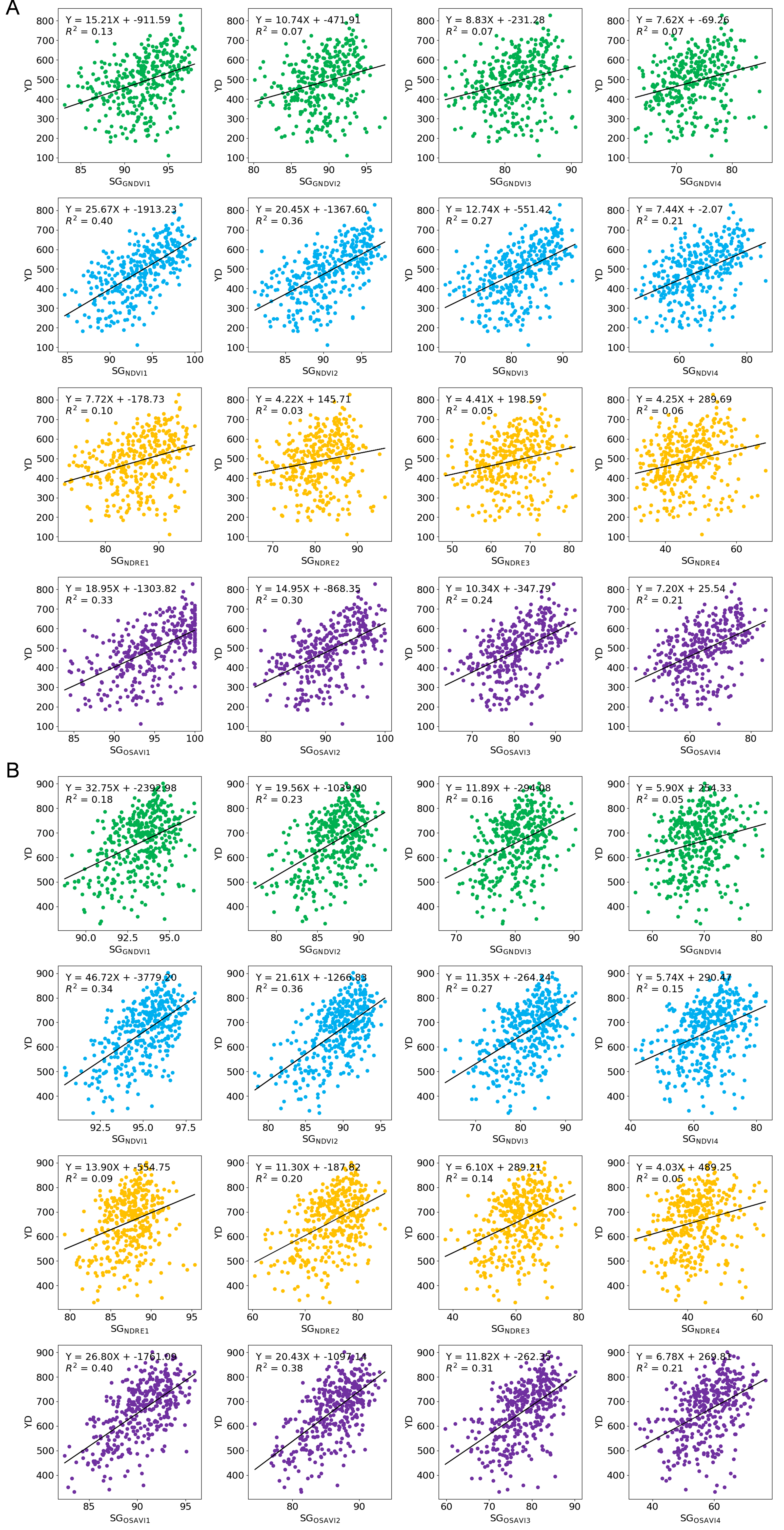


**Fig. S6** Regression results of SG_GNDVI_, SG_NDVI_, SG_NDRE_, and SG_OSAVI_ at four stages (S1 to S4) with yield in the 2021-2022 (A) and 2021-2022 (B).





**Fig. S7** Manhattan plots of SG_NDVI1_, SG_NDVI2_, SG_NDVI3_, and SG_NDVI4_ in 2020-2021 (A-D, respectively), and 2021-2022 (E-H, respectively).





**Fig. S8** Manhattan plots of SG_GNDVI1_, SG_GNDVI2_, SG_GNDVI3_, and SG_GNDVI4_ in 2020-2021 (A-D, respectively), and 2021-2022 (E-H, respectively).





**Fig. S9** Manhattan plots of SG_NDRE1_, SG_NDRE2_, SG_NDRE3_, and SG_NDRE4_ in 2020-2021 (A-D, respectively), and 2021-2022 (E-H, respectively).





**Fig. S10** Manhattan plots of SG_OSAVI1_, SG_OSAVI2_, SG_OSAVI3_, and SG_OSAVI4_ in 2020-2021 (A-D, respectively), and 2021-2022 (E-H, respectively).


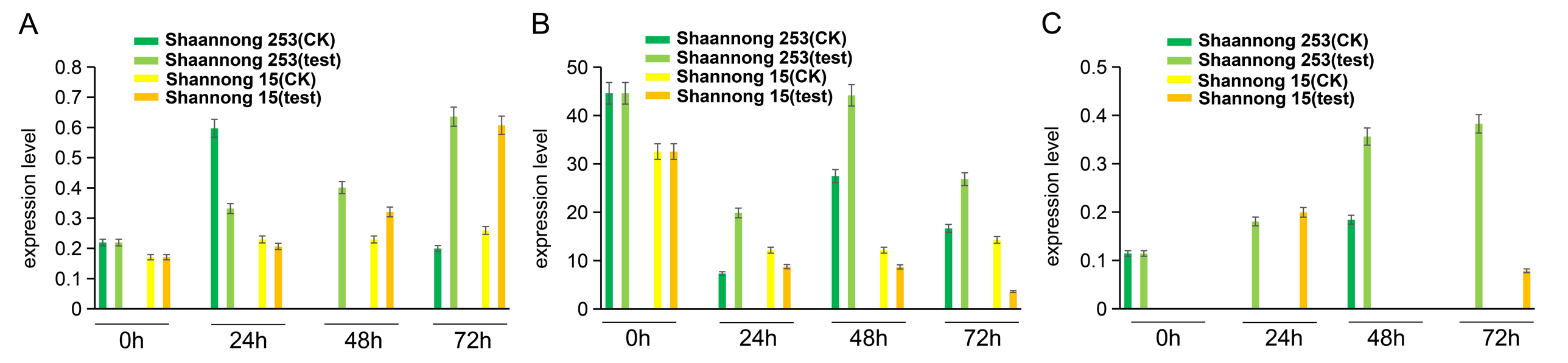


**Fig. S11** Expression profile of *TraesCS6B03G0356400* (A), *TraesCS2B03G1299500* (B), and *TraesCS2A03G1081100* (C) at different times under heat stress 10 days after anthesis in the flag leaf. Shaannong 253 and Shannong 15 are SG material and non-SG material, respectively. “CK” indicates growth in the greenhouse at 22℃ for 16 hours and 18℃ for 8 hours; “test” indicates growth in the greenhouse at 38℃ for 8 hours and 24℃ for 16 hours.


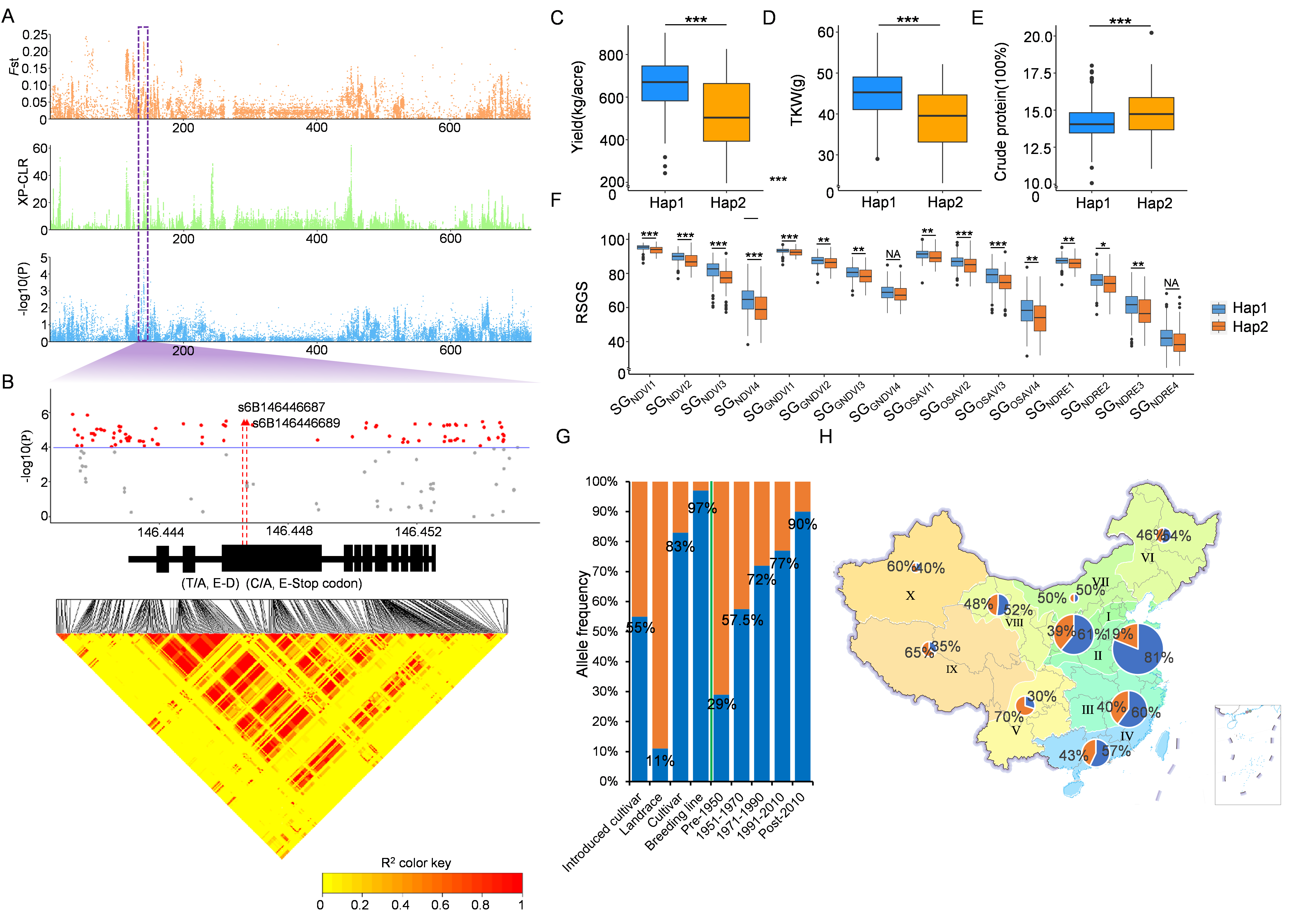


**Fig. S12 Variation in *TraesCS6B03G0356400*.** (A) *F*st, XP-CLR, and associated significant signals on chromosome 6B. The purple rectangle represents the target associated signal position on chromosome 6B. (B) Local Manhattan plot (top) and LD heat map (bottom) surrounding *TraesCS6B03G0356400*. The purple line indicates the significance threshold (−log10[*P*-value] = 4.0). Two red triangles represent two variations in *TraesCS6B03G0356400*. The red color indicates strong LD with the SNP. (C-F) Distribution of the two haplotypes corresponding to yield, thousand kernel weight, crude protein, and RSGS. p-values were calculated using two-tailed t-tests (*, *P* < 0.05; **, *P* < 0.01; ***, *P* < 0.001; NS, not significant). (G) Percentages of haplotypes in different wheat categories (left of the green line) and breeding periods (right of the green line). Hap1 is represented by blue, and Hap2 is represented by orange. (H) Frequencies (%) of haplotypes in different wheat zones. Circle size represents population size; Hap1 is represented by blue, and Hap2 is represented by orange.


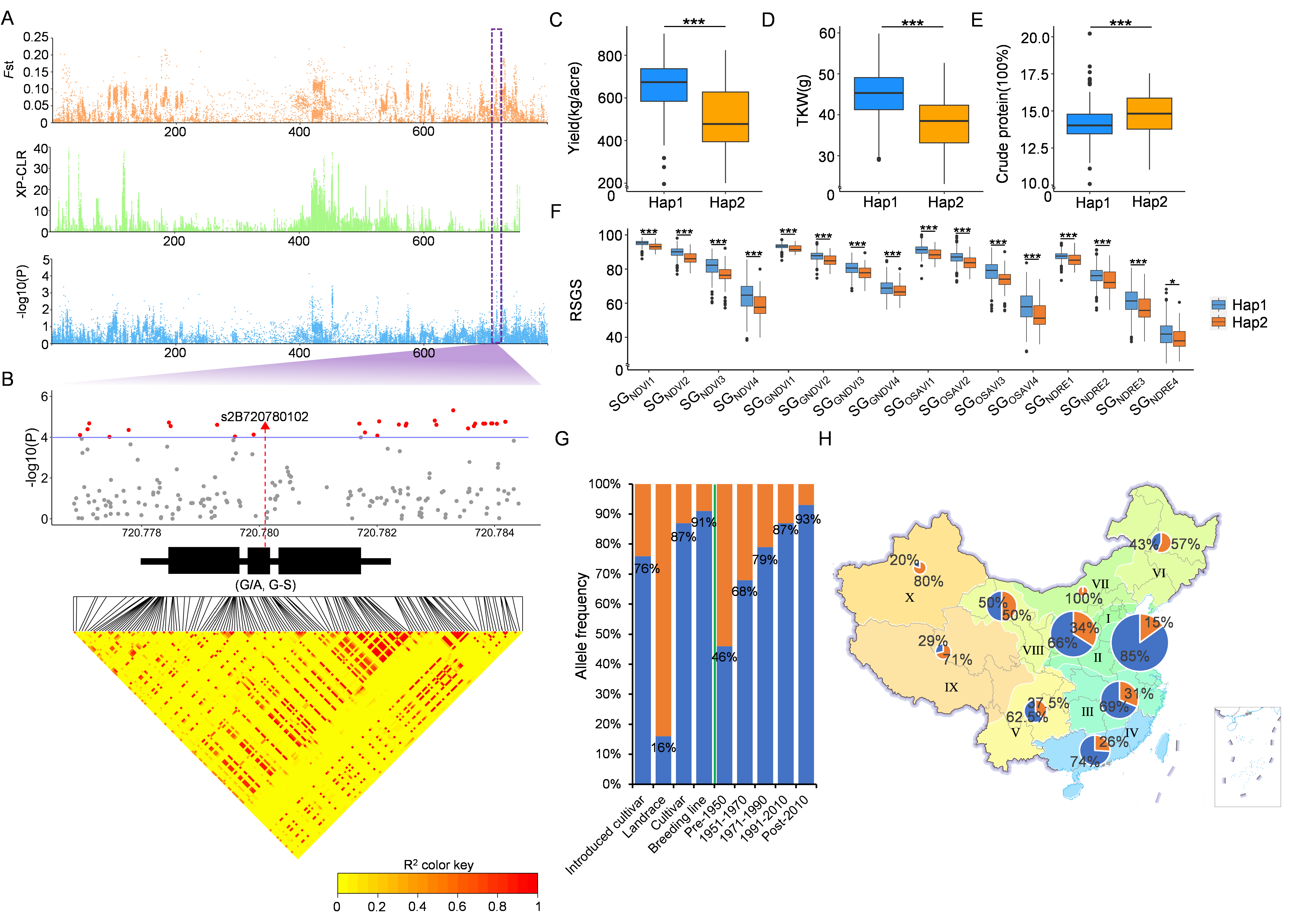


**Fig. S13** **Variation in *TraesCS2B03G1299500*.** (A) The *F*st, XP-CLR, and associated significant signals on chromosome 2B. The purple rectangle indicates the target associated signal position on chromosome 2B. (B) Local Manhattan plot (top) and LD heat map (bottom) surrounding *TraesCS2B03G1299500*. The purple line represents the significance threshold (−log10[*P*-value] = 4.0). The red triangle represents one variant in *TraesCS6B03G0356400*. The red color highlights strong LD with the SNP. (C-F) Distribution of the two haplotypes corresponding to yield, thousand kernel weight, crude protein, and RSGS. *p*-values were calculated using two-tailed t-tests (*, *P* < 0.05; **, *P* < 0.01; ***, *P* < 0.001; NS, not significant). (G) Frequencies (%) of the two haplotypes in different categories (left of the green line) and breeding periods (right of the green line). Hap1 is depicted in blue, and Hap2 is depicted in orange. (H) Percentages of the two haplotypes in different wheat zones. Circle size indicates the number of cultivars; Hap1 is represented by blue, and Hap2 is represented by orange.


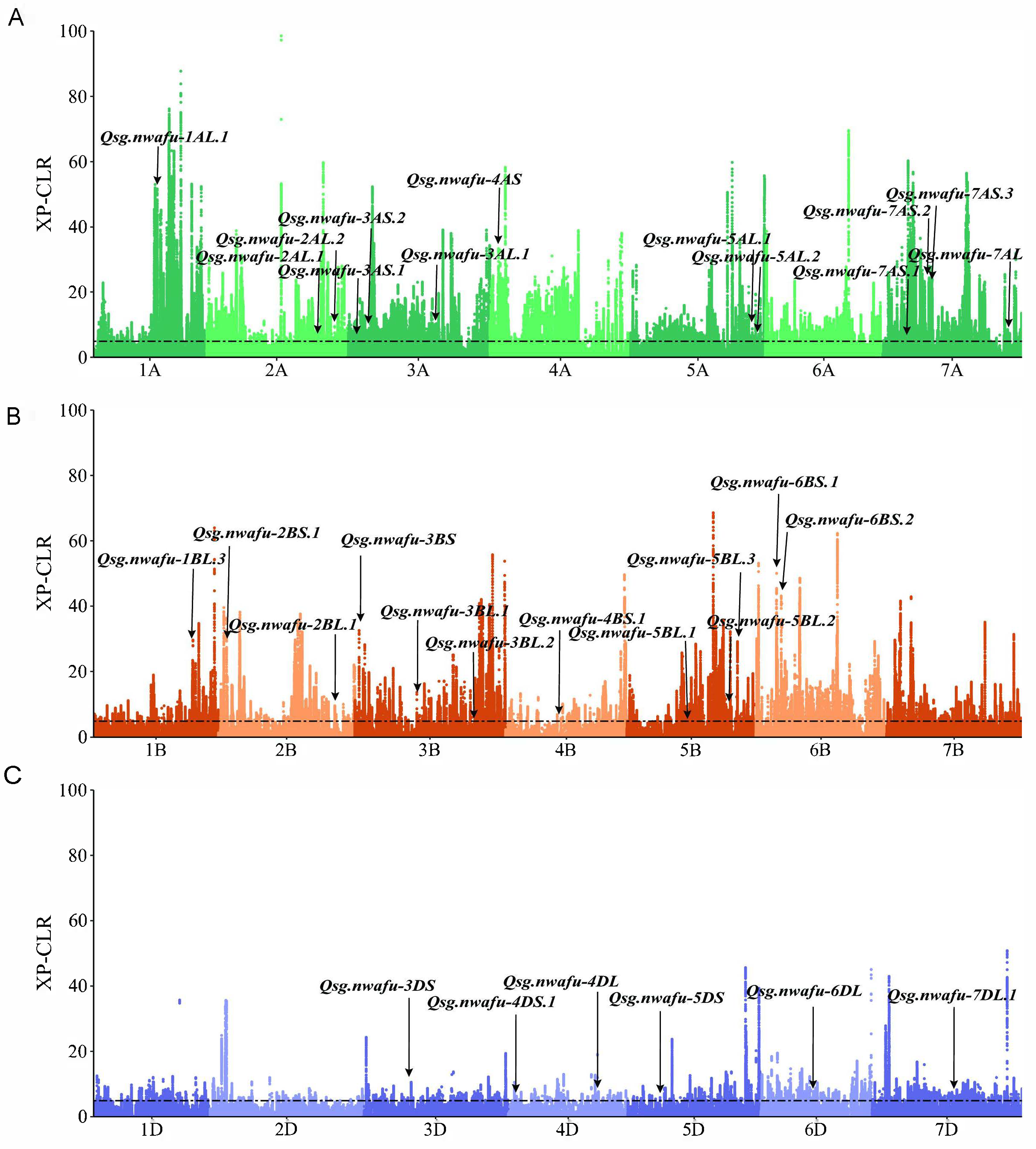


**Fig. S14** **Distribution of XP-CLR scores (Pre-1970 versus Post-1970) for 21 wheat chromosomes.** The SG QTL detected in this study based on their XP-CLR scores are shown. The genome-wide threshold was defined by the top 5% of values.
